# Exploring burnout and staff turnover among acute care nurses and physicians during the COVID-19 pandemic: Insights from a qualitative assessment

**DOI:** 10.1371/journal.pone.0319390

**Published:** 2025-06-04

**Authors:** Rachel L. Snyder, Katelyn A. White, Laura E. Anderson, Ronda L. Cochran

**Affiliations:** Division of Healthcare Quality Promotion, Centers for Disease Control and Prevention, Atlanta, Georgia, United States; Sao Paulo University Faculty of Medicine: Universidade de Sao Paulo Faculdade de Medicina, BRAZIL

## Abstract

**Introduction:**

Healthcare worker burnout and turnover are urgent issues that negatively impact workers and have the potential to impact patient care. To highlight potential factors leading to burnout and turnover among healthcare workers, focus groups were conducted with nurses and physicians who previously worked in acute care settings during the COVID-19 pandemic but have since left their careers providing direct patient care.

**Methods:**

During May and June of 2023, voluntary focus groups were held over Zoom with acute care nurses and physicians who left their careers providing direct patient care. The focus groups each lasted approximately 30 minutes and included 8 or 9 participants. Open-ended prompts used to guide the discussions focused on what led participants to leave their positions and what would need to change for them to return to a role providing direct patient care. The discussions were coded by a team of coders, and then codes were grouped into corresponding themes. Participant demographics were summarized by role.

**Results:**

The 49 focus group participants included 24 physicians (49%) and 25 nurses (51%). Seven convergent themes emerged during the discussions, including the mental toll of their positions, inadequate staffing, the current state of the healthcare system overall, the ways that the COVID-19 pandemic specifically made their jobs harder, support from facility administration, respect and value for their positions, and pay and incentives.

**Conclusions:**

Focus group discussions held with physicians and nurses highlighted multiple factors that led to their decisions to leave their careers providing direct patient care. While more research is needed on the efficacy of specific interventions to address the issues highlighted by participants, various tools and resources are available from multiple organizations to begin to support healthcare facilities in improving the experiences of healthcare workers with the goal of preventing burnout and turnover.

## Introduction

Burnout among healthcare workers (HCWs) has increasingly become an urgent issue in various healthcare settings since the beginning of the COVID-19 pandemic. Burnout includes feelings of exhaustion, cynicism towards one’s work, and reduction of effectiveness in performing professional tasks [1]. Studies have shown that burnout and poor mental health in HCWs has increased since prior to the pandemic [2] and may lead to an increase in staff turnover [3–5]. Identifying and addressing factors that influence burnout in healthcare settings is a priority for the United States Surgeon General [6] and working to prevent and address burnout and turnover in HCWs is especially important, as burnout and turnover may also impact the quality of care provided to patients and lead to billions of dollars in cost to an already stressed healthcare system [7–9].

While many studies have focused on burnout and turnover among HCWs since the COVID-19 pandemic [10–14], literature focusing on the experiences and voices of HCWs who have chosen to leave their positions providing direct patient care is limited. The goal of this assessment was to better understand factors that may lead to burnout and turnover in HCWs through focus groups held with acute care physicians and registered nurses (RNs) who have left their positions providing direct patient care.

## Methods

Physicians and RNs who worked in an acute care hospital during the COVID-19 pandemic but who underwent a career change and now no longer work providing direct patient care were recruited for the voluntary focus groups by Applied Memetics, LLC [15]. This assessment focused on the experiences of those who decided to change or leave their careers, as exploring the perspectives of those who have made the decision to leave their roles may provide important insights into motivations, stressors, and possible interventions to address burnout and turnover. The focus groups took place in May and June of 2023 over Zoom and were homogenous by participant role. Each focus group lasted approximately 30 minutes and consisted of eight or nine participants who were compensated for their time. A trained moderator conducted the focus groups [RC] using open-ended prompts including: “Why did you leave your job providing direct patient care in an acute care hospital?”, “What factors led you to make this decision?” and “What would need to change for you to return to providing direct patient care?”.

Each focus group was recorded with participant permission, transcribed, and anonymized. The transcriptions were then coded as a group by a team experienced in qualitative coding using an immersion-crystallization technique with the qualitative data organized in Microsoft Excel [RS, LA, KW, RC] [16]. Group discussion was used to achieve consistency in assigned codes, which were then categorized into corresponding themes across roles. This activity was reviewed by the CDC and was conducted consistent with applicable federal law and CDC policy (See, e.g., 45 C.F.R. part 46, 21 C.F.R. part 56; 42 U.S.C. §241(d); 5 U.S.C. §552a; 44 U.S.C. §3501 et seq.). This project was exempt from institutional review board requirements as determined by CDC’s National Center for Emerging and Zoonotic Infectious Diseases human subjects’ advisor. Participants provided verbal consent prior to the start of the focus groups. Participants were told that their participation was voluntary, and that they may withdraw from the focus group at any time without ramifications. Verbal consent to proceed by introduction of first name (deidentified after transcription) was collected for every participant.

## Results

A total of 49 HCWs participated in the focus groups, including 24 physicians and 25 RNs who previously worked in acute care settings, but no longer work providing direct patient care (Table 1). The majority of physicians previously worked in the Emergency Department (79%), while 44% of RNs previously worked in the Intensive Care Unit and 40% previously worked in the Emergency Department. Over 75% of participants’ new roles were still in the healthcare field, but no longer providing direct patient care to patients (e.g., now working in an education or research focused role).

**Table 1:** Participant Demographics.

	Physicians (N = 24)		Nurses (N = 25)		Overall (N = 49)	
	N (%)		N (%)		N (%)	
**Unit Type in Previous Role**						
Emergency Department	19	(79%)	10	(40%)	29	(59%)
Intensive Care Unit	2	(8%)	11	(44%)	13	(27%)
Med-surg Unit	1	(4%)	3	(12%)	4	(8%)
Trauma Unit	1	(4%)	0	(0%)	1	(2%)
General Medicine Unit	1	(4%)	0	(0%)	1	(2%)
Post Anesthesia Care Unit	0	(0%)	1	(4%)	1	(2%)
**New Role Type**						
Still in healthcare, but no longer providing direct patient care	19	(79%)	19	(76%)	38	(78%)
No longer working in the healthcare field	3	(13%)	3	(12%)	6	(12%)
Retired	2	(8%)	3	(12%)	5	(10%)
**Participant Time Zone**						
Eastern	12	(50%)	14	(56%)	26	(53%)
Central	6	(25%)	5	(20%)	11	(22%)
Mountain	0	(0%)	1	(4%)	1	(2%)
Pacific	6	(25%)	5	(20%)	11	(22%)

When focus group participants were asked to discuss what factors led to their decision to leave their career providing direct patient care and what would need to change for them to return to a direct patient care role, seven convergent themes emerged from across the discussions (Table 2). Convergent themes included the mental toll of their positions, inadequate staffing, the current state of the healthcare system overall, the ways that the COVID-19 pandemic specifically made their jobs harder, support from facility administration, respect and value for their positions, and pay and incentives.

**Table 2:** Convergent Themes Expressed Across Focus Group Discussions.

Theme	Participants Who Expressed Theme (N = 49)
	N (%)
Theme 1: Mental Toll	44 (90%)
Theme 2: Inadequate Staffing	36 (73%)
Theme 3: State of the Healthcare System	35 (71%)
Theme 4: Challenges Specific to the COVID-19 Pandemic	35 (71%)
Theme 5: Support from Facility Administration	32 (65%)
Theme 6: Respect and Value	19 (39%)
Theme 7: Pay and Incentives	17 (35%)

### The Mental Toll

Across the focus groups, 90 percent of the participants discussed the mental toll their direct patient care roles placed on them. The participants shared how the amount of death experienced during the pandemic affected their mental health. In the words of one physician, “*it was the mental fracturing. It was the first time in my life where you could feel the amount of pain and the emotional weight walking into your shift every day.*”

While prompts asked during the focus groups did not specifically mention burnout in order to limit bias, over 30% of the respondents independently used the word burnout to describe why they left their positions, with RNs discussing that they left their job due to “*caregiver burnout… dreading the alarm going off, dreading the sun coming through the window because you knew it was going to be more of the same*” and that “*it just got to a point that I got so stressed and burned out and anxious that I wasn’t sleeping. I wasn’t eating.*” The feelings of burnout described by the participants were not limited to the COVID-19 pandemic, with one physician expressing “*I think I knew before I finished residency that I was burning out. I was hoping the light at the end of the tunnel would be that going into private community practice would rejuvenate me. That just was not the case. The reality of practicing emergency medicine did not match the idea of what it could be or what it should be*.”

In addition to burnout, participants also described the moral injury and moral distress experienced from not being able to provide quality care to their patients, including one nurse expressing “*it was really frustrating to not be able to provide the care that I wanted to my patients because I didn’t have resources or the time to give that care, the moral distress.”* In the words of one physician*, “It was moral injury but also fatigue. When I take care of a patient, I give my heart and soul. I was burned out. I wasn’t taking care of myself.”*

### Inadequate staffing

Staffing was the next most convergent theme which emerged during the focus group discussions. Both physicians and RNs expressed that the inadequate amount of staffing impacted their decisions to leave their positions and that staffing would need to improve for them to return to providing direct patient care. One RN discussed that *“staffing has always been an issue, but it was very much an issue during COVID.*” A physician described being “*one of the only full-time doctors. A lot of my colleagues who were a little bit older stopped seeing patients clinically for a very long period of time, so it fell to me and some of the junior peers that I have. I was overrun.*” The physicians and RNs also discussed the need for more support staff in their hospitals, with one physician describing the moment they knew they would leave their position “*when there was no one available and I had to carry someone who was blue from their car onto a stretcher. There was no one around, no nurses, no tech, nothing.”*

The focus group participants also thought that management’s responses to fluctuating patient census levels in their facilities often exacerbated staffing issues, including one physician who described how “*when COVID hit they streamlined our hours… but when volumes returned to their previous levels and beyond, they never subsequently adjusted our staffing. So, we had gone from what was considered over staffed to working completely understaffed.”* Many of the RNs described how travel agency nurses who came to the facility to relieve staffing problems still led to a burden on remaining full time staff members, and that full time staff were the ones “*taking the brunt of the work, constantly having to take these severely ill patients because you can’t get someone trained up on how your facility treats them fast enough to utilize [travelers] before they are moving on to something else*.” Several RNs also described being sent to units they were not accustomed to working due to staffing problems, including “*I was getting floated constantly*” and “*nurses would get pulled to the ICU when they aren’t an ICU nurse*.”

The participants offered a few suggestions for what could improve staffing for frontline personnel in acute care hospitals. Several RNs felt “*hospitals need to be better with retention instead of blowing all this money on travelers. Instead, put that money towards retention. Bonuses to keep the existing staff there. We need a reason for those of us who have been around to stay.”* Many RNs in the focus groups also called for *“mandated nurse-to-patient ratios. That would be a big factor for me going back to bedside.”* In the words of one RN, “*the number one thing for me would be better staffing ratios. When you have unsafe staffing ratios, and you have nurses who are picking up 2 or 3 extra shifts a week, it contributes to burnout. We had new grad nurses that started right before the pandemic and a year in they were completely burned out on nursing. No career should burn you out that fast.”*

### State of the healthcare system

Throughout the focus groups, many participants described leaving their positions due to an overarching dissatisfaction with aspects of the way the healthcare system is run, expressing that “*It is unbelievable the state medicine is in currently.”* Both RNs and physicians described lack of autonomy and patient care decisions being dictated by those in administrative positions. In the words of one physician, “*110% there needs to be de-Corporatization of healthcare. This is not the medicine I wanted to go into. There has to be a sense of autonomy. That is what physicians are trained to do. We are trained to be in charge of our patients, to be our patients’ advocates, and to be in charge of ourselves…. our current generation and our predecessors have allowed everyone else who has no idea what is going on as far as healthcare at the bedside to run everything.”* One RN expressed that one thing that would need to change for her to return to patient care was *“the decision making by administration. Decisions were coming down where it was so clear no one was asking the people who were actually doing the job and taking care of all the patients.*”

The participants also described feelings that profits were being prioritized over people, with one physician expressing that their facility “*would not authorize certain medications because it was too expensive, saying that you are going to bankrupt the hospital. It was a disgusting side of medicine by administration. A lack of support and trying to influence care based on financials*” and that healthcare companies *“are beholden to shareholders to gain shareholder value quarter to quarter… how can you increase profits when the insurance companies have a set rate? You can’t just start charging CMS more money tomorrow. The only way to make more money is by cost cutting. That increases revenue quarter after quarter, but cost cutting is at the cost of care to our patients. Care to the staff.”* Other physicians described how *“the business side of medicine is unfortunately not centered on healthcare providers and not centered at the nursing level or at the patient care and family level. So that’s when you feel powerless, and then you start seeing the quality of care being compromised. And then you start looking for the exit”* and that they *“don’t think it’s fair to put myself or my patients at risk by putting profits over people.”*

The participants also discussed how the increased use of contract management groups, demands of electronic charting and metrics, and the lack of comradery in healthcare today impacted their career decisions. One physician described how they *“used to love walking into my shift and the relationships and the rapport you built with your support staff because you have to have a strong team to care for anything that walks through the door. And honestly, I don’t know how to get that back. I feel now with all the metrics that are involved and how easily we are replaced and how we aren’t even a hospital employee, I feel that not only as an MD, but also as a person, I have no rights really at the hospital. Nothing. No matter what I say to administration or do, it really doesn’t matter.”* Another physician expressed that “*I would never work for a contract management group again*.” RNs suggested cutting down on *“the enormous amount of electronic charting... Am I charting or am I taking care of the patient? What do you want me to do first?”* and that *“we have to go back to focusing on the patients.”*

When describing what would need to change for them to return to providing direct patient care, one physician discussed the need for “*big changes to metrics, which would lead to big changes in staffing… hopefully, those two things would also correlate with a change in value and ethics, which are missing almost entirely right now from the way healthcare is run.”* Additionally, several participants described that their interactions with patients have changed for the worse, with one physician expressing that they “*got tired of the abuse from patients. The lack of respect that most patients have when they come into the ED. It’s just insane. Emergency medicine was bad before, but now emergency medicine is like going into customer service. Like you are going out to eat at a restaurant and there are all these horrible metrics we have to meet, like door to doc times, transfer rates. It is just a mess*.”

Several participants worried about the future of healthcare, with one physician stating “*I can’t fathom what healthcare is going to be when we need it. I had 7 years of training and I called it quits... I loved what I did, but was disgusted by the system and the people who were in charge of the system.”* One RN felt that the COVID-19 pandemic “*pushed down the tumbling wall that already was healthcare. We were already stretched too thin before COVID and COVID then pushed it over the edge.”*

### Challenges specific to the COVID-19 Pandemic

In addition to enduring challenges in healthcare, such as staffing and HCW burnout, the participants also discussed how impacts and experiences specific to the COVID-19 pandemic affected their decisions to leave their careers providing direct patient care, including supply shortages, the increased burden of severely ill COVID-19 patients, and the helplessness and uncertainty of treating patients infected with a novel pathogen. One RN described that she had “*been a nurse for 15 years. I didn’t really have any strong desire to leave the bedside, but COVID is what did me in*”, while another expressed that “*it was overwhelming… I was afraid because we didn’t know what was going to happen, the way the virus was mutating. I don’t know if I have it in me anymore to be that much of a first line nurse for another catastrophe like that.*” One physician described how “*for the first time in my career, I had to tell people we don’t know what to do. We’re figuring this out as we go along. It almost felt like I couldn’t be a doctor anymore. They were coming to the ER for help, but the best I could do in many days was a really good guess and I was flipping a coin*.”

Some participants left their careers due to concerns for themselves or their family members contracting COVID-19. One participant described that “*we were newly married and wanting to start a family. With COVID, I was working in direct patient care and we were being told not to bring it home to little ones and the elderly, because there was a lot of negative outcomes happening. So, for me, with our choice to start a family, I needed to get out of the healthcare setting*.” Another RN expressed how “*at the time I had my 88-year-old grandmother who lived with me, my husband has COPD, and I was so terrified I was going to bring it home and get them sick because I didn’t have the resources I needed to take care of my patients safely. I could not do it anymore*.”

Other participants described how misinformation, politicization of the pandemic, and COVID-19 denial from both patients and coworkers impacted their career decisions. One physician discussed that they “*didn’t understand how many of my coworkers didn’t believe in vaccines and didn’t believe in science… I had to take care of four unvaccinated coworkers with COVID who I had to intubate, three of whom subsequently died. So that is when I was done.*” RNs described how patient denial of COVID-19 impacted them by expressing “*there was a lot of COVID denial. We had patients who were about to be intubated looking you dead in the face and saying ‘this isn’t COVID.’ It gets to you after a while. You can only take so much”* and that *“we had patients coming in and refusing to wear [masks] denying the fact that they had COVID symptoms, getting other nurses sick… We lost some of our older staff because patients refused to do the proper thing. Once we went to the funeral, that was all for me.”* Another participant described how *“one of the most frustrating things was the lack of trust that occurs because of politicization of the whole pandemic, which has really taken an effect on everything else. People are looking at you with distrust, they are questioning you, not wanting to go along with your recommendation. It is very frustrating when you are trying to deliver what you are trained to do as a physician, which is to give the best care you can to the patient.”*

### Support from facility administration

The participants also described how they desired better support from administration in their facilities, including one RN who expressed the need for “*more understanding from administration. That is the biggest thing. I don’t feel nurses think they are supported any longer.”* The participants discussed the importance of a workplace culture that supported staff well-being, with one physician describing how *“there needs to be a different culture of understanding and of accepting and supporting physician well-being. I think a lot of times people expect us to be the superheroes and never take care of ourselves. I need to make sure I am going back to a culture or a system that supports me and not just what I can do.”*

The participants thought that this culture of support could include the flexibility to provide a better work-life balance and administration who listened to the concerns voiced by staff. One physician described how in their facility *“when concerns [were] voiced to administration… I almost felt like I was gaslit. It felt like suddenly there was this stiff upper lip.*” Physicians also described how in order to return to providing direct patient care they “*would have to find something that allowed me the flexibility in my schedule”* and that “*it would have to be a culture of understanding. Physicians need breaks, better balance*.” In the words of one RN, staff “*knew what they needed to do, but we just didn’t have [the support] we needed to do it. You do need breaks during the day. You do need that lunch hour.*” Nurses also described the importance of *“transparency from management and then also the willingness for management to step in. If they have to cover some assignments, that they are willing to. A lot of times management is hesitant to do that. We needed the guidance from them and for them to be that role model*.”

### Respect and value

Another convergent theme which emerged during the focus groups was the need for respect and value for HCWs. Several participants described feeling replaceable by their facilities, including that “*there was a lack of value and respect. I would want to see that change. That we are valued, not so much in payment, but in how much we bring to the table, and not made to feel like we are replaceable”* and also that *“if I’m not going to be valued by others, I am going to value myself.”* One physician described how *“the culture changed a lot over the past 20-30 years to shift away from the physician being a respected professional to being a means to an end.”* To illustrate the lack of respect faced by personnel, one RN described a situation where they *“remember the hospital putting a ‘Heroes work here’ sign on the very same parking garage that we weren’t allowed to park in... It was just another slap in the face.”*

### Pay and incentives

The participants also discussed how pay and incentives impacted their decisions to leave their positions providing direct patient care. In the words of one RN, facilities needed to *“pay us what we are worth. Pay us what we are sacrificing. Don’t just say that we should be doing it because that’s what nursing is. I was very glad to get out of there*.” Another RN expressed the need for *“better wages for the nurses… bonuses. Because if you offer a decent wage for your employees, you can then recruit experienced nurses to your unit and keep them around so you can build that family, that camaraderie.”*

Several emergency department physicians described that they were not compensated fairly compared to other units in their hospitals, including that their hospital told them they were thinking about *“decreasing [our] pay… And it was just crazy because everywhere else in the hospital was getting bonuses. And we are here working the front lines. We are the ones supplying the hospital with their patients… It is horrible how we were treated.”*

Both physicians and RNs described the pay disparity with travel nurses which occurred in their facilities, including that they *“are bringing in travel nurses who are making 3-4x what we made and were expecting us to do the same job. That’s just not fair”* and that healthcare systems *“were more than happy to throw a lot of money at traveling nurses, but there were times when seasoned, well-trained doctors were making less than the traveling nurses.”* One RN described feeling *“guilty wanting the money when I should be there for the people. So, it really was this love hate relationship with the job… I have no desire to take care of people full time [anymore] and money is a motivator. I’ve gotten to the point where I am okay with saying I want more money.”*

## Discussion

Focus group discussions held with RNs and physicians who left their careers providing direct patient care illustrated that multiple factors impact the wellbeing of HCWs and HCW turnover. While addressing and preventing HCW burnout and turnover will require interventions at the individual, facility, and system levels, existing resources are available from multiple organizations to assist facilities in reducing and preventing HCW burnout and turnover and providing a healthier and safer environment for HCWs and patients.

Focus groups in this assessment aligned with previous studies in highlighting the mental toll placed on HCWs, including the potential impact of HCW burnout on turnover [2–5,12]. Two interventions discussed in the literature to address the mental toll that a career in healthcare can place on workers include creating peer support programs amongst HCWs [17,18] and removing barriers for HCWs to receive mental health support [19,20]. Both the American Medical Association (AMA) and the American College of Physicians (ACP) have developed resources related to peer support programs for physicians [21,22], while the American Nurses Foundation Well-Being Initiative also includes resources for live peer support for nurses [23].

Although peer support programs may help address the mental toll placed on HCWs, it is also important that clinical mental health care and treatment is provided when needed, and that HCWs feel comfortable accessing this care [24]. Action three of the National Institute for Occupational Safety and Health (NIOSH) Impact Wellbeing Guide includes resources for auditing and updating facility credentialing application questions which may influence whether HCWs seek mental health care, including questions referencing past mental health treatment which may be stigmatizing [25]. The ACP and the Dr. Lorna Breen Heroes’ Foundation have both also developed toolkits related to updating credentialing application questions related to mental health care [26,27].

The participants in the focus groups also discussed the impact of understaffing on their decision to leave their positions as well as their desire for better standards for staffing ratios. This theme aligns with previous literature related to burnout and turnover among HCWs [28–31]. While more research is needed related to appropriate staffing ratios in healthcare, George Washington University’s Workplace Change Collaborative includes strategies for health organizations to work towards safer and appropriate staffing, including a compilation of resources for creating organizational staffing policies in healthcare and using technology to improve workflows [32]. The AMA has also developed resources for reducing unnecessary burdens on clinicians, including strategies related to increasing efficiency of electronic health records, which may help to address some of the concerns related to the current state of the healthcare system which was expressed by participants in the focus groups [33].

Another theme found both in the literature and the focus groups in this assessment was the potential impact of support from facility administration on HCW burnout and turnover [31,34–36]. In order to ensure that HCWs feel supported by their organization and administrative leadership, showing a commitment to the well-being of HCWs at the top levels of senior leadership is important. The Mayo Clinic Strategies to Reduce Burnout includes information for building a business case for well-being programs to assist with gaining leadership buy-in for efforts related to HCW well-being [37]. Additionally, the AMA provides resources on creating a dedicated Chief Wellness Officer position to advocate for and coordinate initiatives focused on preventing burnout and promoting worker wellbeing [38] and the second action of the NIOSH Impact Wellbeing Guide illustrates roles for creating a wellbeing team in facilities [39]. The involvement of unit-level champions for wellbeing may also help HCWs feel supported in their organizations [40]. The focus group participants also described feeling that they were replaceable and were not respected or valued by their facilities. Various studies have also highlighted the importance of HCWs feeling valued and the potential impact of meaningful recognition for HCWs [41,42]. The American Association of Critical-Care Nurses includes Meaningful Recognition as an essential aspect of creating a healthy work environment and provides resources for appropriately recognizing HCWs [43].

Many participants in the focus groups discussed the specific challenges posed by the COVID-19 pandemic, such as a lack of resources, which aligned with those in the literature [44,45]. However, their responses also aligned with existing literature in underscoring how the challenges of COVID-19 exacerbated problems that were already present in healthcare systems [28,46], thus illustrating the importance of working to address the concerns of healthcare workers beyond those posed solely by the pandemic, in addition to learning from the pandemic experience itself.

Although more research on the efficacy of individual interventions to reduce HCW burnout and turnover is needed, many organizations are continuing to develop resources and strategies to assist healthcare facilities in supporting HCWs, which is an important step in beginning to address the concerns highlighted by HCWs in this assessment as well as in the literature. This qualitative assessment was not without limitations. Participants in the focus groups represented a convenience sample and may not be representative of the larger population of RNs and physicians nationwide. Responses in this assessment may have been subject to self-report as well as recall bias. Due to the limitations of this qualitative methodology, the findings presented from this assessment may not be generalizable. Additionally, while demographic data available for participants in this assessment was limited, actions to further evaluate HCW turnover and burnout among various demographic groups are important for creating a workplace culture that is supportive of all HCW well-being [47].

This qualitative assessment has highlighted the voices of RNs and physicians who made the decision to leave their career providing direct patient care, illustrating the motivations that HCWs have when making such decisions. Addressing factors which may impact HCW burnout and turnover is important for the safety of workers as well as patients [7]. Therefore, incorporating HCW well-being and burnout prevention activities into larger quality improvement efforts is an important step in working to prevent HCW turnover and improve the quality and safety of healthcare overall [37,48].
